# Polymeric nanocapsules with up-converting nanocrystals cargo make ideal fluorescent bioprobes

**DOI:** 10.1038/srep29746

**Published:** 2016-07-13

**Authors:** U. Bazylińska, D. Wawrzyńczyk, J. Kulbacka, R. Frąckowiak, B. Cichy, A. Bednarkiewicz, M. Samoć, K. A. Wilk

**Affiliations:** 1 Department of Organic and Pharmaceutical Technology, Faculty of Chemistry, Wrocław University of Science and Technology, Wybrzeże Wyspiańskiego 27, 50-370 Wroclaw, Poland; 2 Advanced Materials Engineering and Modelling Group, Faculty of Chemistry, Wrocław University of Science and Technology, Wybrzeże Wyspiańskiego 27, 50-370 Wroclaw, Poland; 3 Department of Medical Biochemistry, Wroclaw Medical University, Chałubińskiego 10, 50-368 Wroclaw, Poland; 4 Institute of Low Temperature and Structure Research, PAS, Okólna 2, 50-422 Wroclaw, Poland; 5 Wroclaw Research Center EIT+, Stablowicka 147, 54-066 Wroclaw, Poland

## Abstract

An innovative approach for up-converting nanoparticles adaptation for bio-related and theranostic applications is presented. We have successfully encapsulated multiple, ~8 nm in size NaYF_4_ nanoparticles inside the polymeric nanocarriers with average size of ~150 nm. The initial coating of nanoparticles surfaces was preserved due to the hydrophobic environment inside the nanocapsules, and thus no single nanoparticle surface functionalization was necessary. The selection of biodegradable and sugar-based polyelectrolyte shells ensured biocompatibility of the nanostructures, while the choice of Tm^3+^ and Yb^3+^ NaYF_4_ nanoparticles co-doping allowed for near-infrared to near-infrared bioimaging of healthy and cancerous cell lines. The protective role of organic shell resulted in not only preserved high up-converted emission intensity and long luminescence lifetimes, without quenching from water environment, but also ensured low cytotoxicity and high cellular uptake of the engineered nanocapsules. The multifunctionality of the proposed nanocarriers is a consequence of both the organic exterior part that is accessible for conjugation with biologically important molecules, and the hydrophobic interior, which in future application may be used as a container for co-encapsulation of inorganic nanoparticles and anticancer drug cargo.

We present here a new type of biocompatible luminescent probe based on the concept of encapsulation of multiple up-converting lanthanide-doped nanoparticles (UCNPs) inside a single multifunctional polymeric nanocontainer. Rapid development of wet chemistry synthetic routes in the last decade has provided the possibility to obtain colloidal NPs of various chemical character, with engineered morphology, crystal structure and also with precisely designed optical, electrical or magnetic properties^1^,^2^,^3^. The versatility of such nanomaterials has opened new perspectives for real life applications, with great attention being paid to bioimaging and nanomedicine^4^. However, as the wet chemistry synthesis techniques usually yield hydrophobic NPs, the efficient and straightforward surface hydrophilization remains the main challenge. Several strategies of colloidal NPs surface functionalization towards bioapplications exist, and in the case of UCNPs are limited mainly to: ligand exchange, ligand attraction, ligand oxidation, layer-by-layer assembly, encapsulation in silica shell and host-guest self-assembly^5^. Those techniques are, however, time consuming, complicated and require further steps for conjugation of NPs with different functional components such as therapeutic agents or targeting groups. It needs to be stressed out, that while the use of various NPs as markers for fluorescence imaging is currently well established, there is still need for “smart” theranostic agents, which are nano-engineered to combine both therapeutic and diagnostic functions. Recently, G. Jalani *et al*.^6^ presented very interesting results on near-infrared (NIR) switched drug delivery with the use of UCNPs coated with hydrogel. We are, however, proposing a new approach for the construction of theranostic agents, which goes beyond the archetype of a single NP being functionalized with ligands or functional molecules, and envisage various types of nanocontainers as universal therapeutic and diagnostic cargo carriers. In fact, polymeric nanocarriers with sizes lower than 200 nm have been receiving increasing attention as containers for hydrophobic NPs encapsulation, providing good solubility, high long-term stability and loading capacity as well as ability of sustained release of active cargo^7^,^8^,^9^. There are numerous biological benefits of simultaneous multiple NPs encapsulation including (i) their improved and prolonged biodistribution in the body; (ii) enhanced biocompatibility and (iii) better internalization by tumor cells^10^,^11^. The structure and surface of polymeric nanocarriers can be further intentionally designed for specific applications, including functionalization with folic acid, polyethylene glycol or bio-compatible polyelectrolyte, which allows to obtain a wide range of functionalities of the nanocontainers. As the polymeric nanocapsules act as the targeting factor, the active cargo composed of the NPs and other species can be designed for bioimaging and/or therapeutic applications, including cytostatic drugs distribution visualization or photodynamic cancer treatments^12^,^13^. In that way, the cumbersome functionalization of individual NPs surfaces is omitted, and the scenario of simultaneous organic and inorganic species co-encapsulation allows for designing specific interactions, such as efficient Förster Resonance Energy Transfer (FRET) between hydrophobic NPs and photoactivated drugs (e.g. porphyrins) which may allow for FRET based treatments and sensing^14^,^15^.

Encouraged with our recent results in encapsulation of colloidal quantum dots (QDs), and their application as two-photon markers in bioimaging^16^,^17^, we have decided to use a similar approach for another type of inorganic NPs with great potential for bioapplications – up-converting NaYF_4_ NPs. Unlike in the case of QDs or organic dyes, which can produce blue-shifted emission only through the mechanism of two-photon absorption requiring high light intensities, the visible emission from up-converting NaYF_4_ NPs is obtained under continuous wave, low power laser diode excitation from NIR region^18^. This lowers the autofluorescence background noise and allows for deep penetration of excitation light into biological tissues. Additionally, co-doping the NaYF_4_ NPs with Tm^3+^ and Yb^3+^ ions allows one to use both excitation (976 nm) and emission (800 nm) from the spectral range considered as “window of optical transparency” of biological tissues^19^. This feature provides high contrast for *in vitro* and *in vivo* bioimaging, including cells and tissue labeling, since the absorption and scattering are significantly reduced in the NIR spectral range^20^. The main benefits from up-converting NaYF_4_ NPs encapsulation are therefore: (i) the increase of the particles hydrodynamic diameter, which increases their retention rate compared to single NPs with sizes around 10 nm, which are efficiently extracted from the body by the urinary system; (ii) prevention of the direct interaction of fluoride and lanthanide ions with proteins and biological molecules, which could disturb the electron/ion cell membrane transport activity causing oxidative damage or lipid peroxidation; and finally (iii) the increase in the cellular uptake by the application of positively-charged biocompatible polymers (e.g. saccharide-based) or polyelectrolytes (PEs) for nanocapsules fabrication.

With this in mind we have designed two types of multifunctional core-shell nanocarriers obtainable by relatively easy, not time-consuming “self assembly” approaches. The strategy applied for fabrication of the NaYF_4_:Tm^3+^,Yb^3+^ NPs-loaded nanocapsules via a two-step process is presented in Fig. 1. For the NPs encapsulation we used the emulsification-solvent evaporation method (Stage I), followed by layer-by-layer (LbL) saturation technique (Stage II). As it has been proved before, the LbL adsorption of PEs on the charged oil-core is one of the most promising techniques for versatile nanocontainers fabrication. Furthermore, LbL assembly allows for engineering their shells on the nano-level, leading to the construction of biocompatible nanocontainers with desired biospecific properties including (i) improved biodistribution via pegylation process; (ii) tumor targeting via functionalization of the top PE layer with ligand of a specific cell receptor overexpressed on tumor cells; (iii) reduced immunogenicity via application of protein- or polysaccharide-based materials^21^. Additionally, the selection of appropriate components for nanocapsule preparation is crucial for achieving a long term stability and biocompatibility of the functional cargo as well as its improved internalization by target tumor cells^22^,^17^. The results of our previous experiments^21^ indicated that the essential prerequisite to construction of long-term container shells is formation of stable interfacial complex between an appropriate ionic surfactant and the first layer of oppositely charged PE.

In our recent papers we proved that dicephalic-type surfactants showed the strongest interactions with investigated (primary sugar-based) polyelectrolytes followed by creation of stable multilayer nanocapsules with appropriate size, high colloidal stability, high loading capacity and controlled permeability. Based on previous results, in the present contribution we propose a representative of environmental friendly cationic dicephalic-type surfactant class, i.e., *N,N*-bis[3,3-(dimethylamine)propyl] dodecanamide dichloride, C_12_(DAPACl)_2_ as a stabilizer of silicone core polymeric template used for the successive LbL PEs deposition of different, i.e, standard (PSS/PDADMAC) and sugar-based (DEX/CHIT) PE shells. Consequently, we have created (Fig. 2) novel custom-designed multifunctional nanocontainers with various PE shells, dedicated for encapsulation of NaYF_4_:Tm^3+^,Yb^3+^ NPs.

## Results and Discussion

### Characterization of NaYF_4_:Tm^3+^,Yb^3+^ - loaded oil-core polyelectrolyte nanocapsules

We have investigated the morphology and crystal structure of as-synthesized NaYF_4_:Tm^3+^,Yb^3+^ NPs intended for the encapsulation experiments by the XRD and TEM techniques. Based on NPs XRD pattern measurements and comparison with the standard (ICSD-60257) one, we have assigned the obtained NaYF_4_:Tm^3+^,Yb^3+^ NPs to pure cubic crystal structure of sodium yttrium fluoride (Fig. S1b). The noticeable broadening of the XRD peaks was related to the small size of NPs, which did not exceed 10 nm. The as-synthesized NaYF_4_:Tm^3+^,Yb^3+^ NPs size was further confirmed by TEM imaging, the studied NPs had spherical shapes with mean sizes below 10 nm, and showed narrow size distribution (Fig. S1a,c). The elemental EDAX analysis also showed expected peaks characteristic for Y, Yb, Na and F elements (Fig. S1d). In the next step, the NaYF_4_:Tm^3+^,Yb^3+^ NPs - loaded nanocapsules were constructed via an emulsification solvent-evaporation method followed by LbL approach with the use of SO, Poloxamer 403 and C_12_(DAPACl)_2_ as the oil-core polymeric template, as well as PSS/PDADMAC and DEX/CHIT as the standard and biodegradable shells, respectively. The NaYF_4_:Tm^3+^,Yb^3+^ NPs were loaded into the multilayer nanocapsules at three different concentrations (0.4–1.5 mg/mL) to select the most stable and optimal system for biological evaluation. The set of formulation conditions for the obtained nanocapsules is shown in Table 1. The mean diameter (D_*H*_), polydispersity index (PdI) and zeta potential (*ζ*) of the nanocapsules were assessed on the day of the oil-core nanocarriers production, and 30 days after their storage at room temperature (see Table 1S in ESI). The formation of the multilayer polyelectrolyte nanocapsule shells by subsequent adsorption of PSS/PDADMAC and DEX/CHIT bilayers on the polymeric oil-core template and the stability of the obtained nanocapsules were confirmed by examining the overall surface charge of the nanocarriers after each PE layer deposition. The surface charge was determined by *ζ*-potential measurements and, as it has been proved by our previous studies, stable nanostructures were obtained when the zeta potential of nanocarriers with the adsorbed polyelectrolyte layer was close to the zeta potential of the same polycation or polyanion in solution^21^. Therefore, the volumes of polyelectrolyte solution used to form each layer of nanocapsules shell were determined experimentally by the evaluation of the results of simultaneous *ζ*-potential potential measurements. Figure S2 in ESI shows the typical dependence of nanocapsules zeta potential on adsorption of the consecutive PE layer on the polymeric core (the final *ζ*-potential values for the obtained nanocarriers are shown in Table 1). The observed regular layer to layer variations of zeta potential from −44 to  + 57 mV for PSS/PDADMAC and from −55 to + 43 for DEX/CHIT PE pairs prove that stable nanocapsule shells were obtained, and no effect of the different NaYF_4_:Tm^3+^,Yb^3+^ NPs concentration on the *ζ* values was observed. Additionally, all the studied samples showed minor variations of the surface charge; in particular the nanocapsules with lower NPs concentrations (systems 1a and 2a) showed the most stable value of the zeta potential after storage in the dark (T = 30 days). Furthermore, the recent data suggest that the positive charge of the nanocapsules can have favorable effect on the extended nanocarrier long-term circulation in the blood stream and improved distribution *in vivo*^23^,^24^. The results of size and polydispersity measurements indicated that the empty and loaded nanocapsules prepared with various NaYF_4_:Tm^3+^,Yb^3+^ NPs concentrations displayed an average size between 110 and 147 nm and PdI of 0.19–0.26. The hydrodynamic dimensions (<150 nm) corresponded well with our data reported for other polymeric nanocarriers loaded by inorganic NPs designed mainly for anticancer and bioimaging applications^17^. As expected, with increase of the NaYF_4_:Tm^3+^,Yb^3+^ NPs concentration the nanocapsules diameter also increases. Furthermore, the most unimodal particle size distribution followed by the long term colloidal stability (at least 30 days of storage in the dark) was achieved for the nanocapsules with lower NaYF_4_:Tm^3+^,Yb^3+^ NPs concentration (systems 1a and 2a) regardless of the type of PE bilayer used.

**Figure 1:**
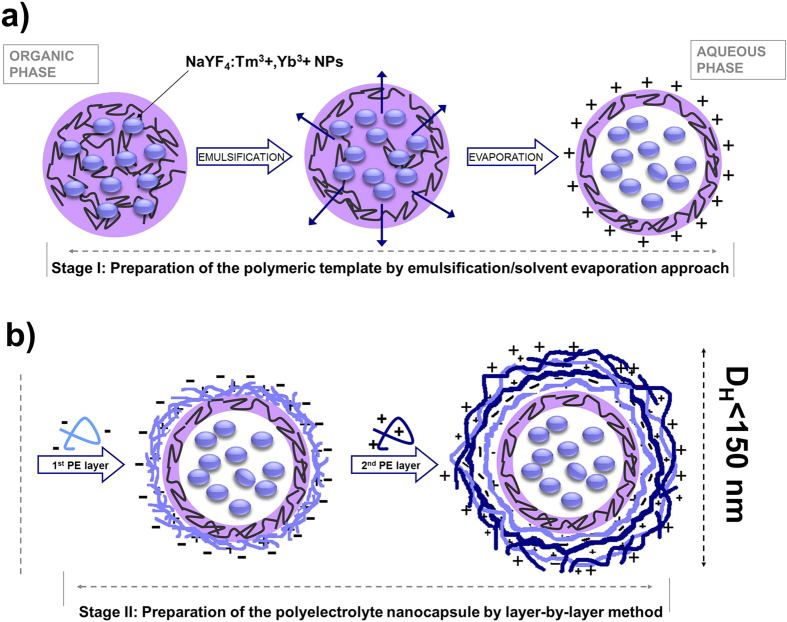
The general idea of the NaYF_4_:Tm^3+^,Yb^3+^ NPs loaded oil-core polyelectrolyte nanocapsules fabrication via two-step process: emulsification/solvent evapoaration (**a**) and LbL (**b**) approach.

The surface morphology of the obtained nanocarriers characterized by two independent techniques: atomic force microscopy (AFM) and transmission electron microscopy (TEM), is compared to the DLS distribution graph, as shown in Fig. 3. The obtained images indicate that the studied multifunctional nanocarriers loaded with NaYF_4_:Tm^3+^,Yb^3+^ NPs appeared well-separated, spherical in shape without significant aggregation, indicating that they were effectively stabilized by the surrounding polyelectrolytes, triblock copolymer and the surfactant shell in aqueous solution. The structuring of NaYF_4_:Tm^3+^,Yb^3+^ NPs inside the nanocapsules, as well as morphology of the nanocapsules itself, was visualized by TEM (Fig. 1b and Fig. S3). It is clearly observed that the NaYF_4_:Tm^3+^,Yb^3+^ NPs with an average diameter of 8 ± 2 nm (visible as dark spots) are formed into larger spherical aggregates, with sizes between 90 nm and 160 nm. Those results were in a good agreement with AFM imaging and DLS spectra. The observed distinct architecture and the lack of free NPs confirm therefore that the NaYF_4_:Tm^3+^,Yb^3+^ NPs were successfully encapsulated in the interior cavities of the nanocapsules, and efficiently protected from leaking out of the oil-core nanocarriers.

Taking into account the mean sizes of (i) single NaYF_4_:Tm^3+^,Yb^3+^ NP and the (ii) NPs-loaded nanocapsule, we have estimated the rough number of NPs in single nanocarrier to be ~2400. The 3D AFM images of the obtained nanocarriers as well as an individual multilayer nanocapsule presented in Fig. 3a, show their semi-smooth surface without certain roughness, although 2D images display relatively smoother surface morphology. The nanocapsules with various types of PE shell did not reveal significant differences in morphology, thus we present a representative image for the NaYF_4_:Tm^3+^,Yb^3+^ NPs -loaded sample with DEX/CHIT shell (system 2c according to Table 1). The average size estimated based on TEM and AFM imaging of the nanocapsules was around 120 nm, which is in good agreement with the values obtained by DLS measurements (Fig. 3c). Thus, we have demonstrated that the obtained nanocapsules exhibit an exceptional nanocontainer structure capable of enclosing diverse inorganic nanomaterial in the oleic interior surrounded by different PE shields.

**Figure 2:**
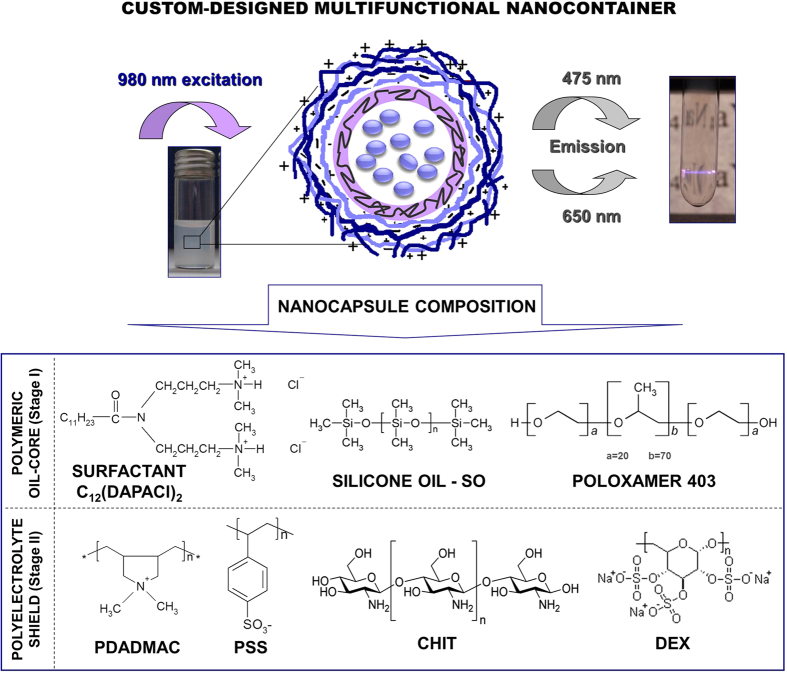
The schematic presentation of the custom-designed nanocontainer loaded with NaYF_4_:Tm^3+^,Yb^3+^ NPs with the structures of the main nanocapsule components.

Besides the time-dependent measurements of the nanocapsules size by DLS, we have assessed the possibility of any destabilization phenomena (aggregation, sedimentation) also forced by possible release or leakage of the encapsulated nanocrystals in the suspensions of the obtained nanocapsules, with the use of Turbiscan^25^,^26^. Because of the extremely high hydrophobicity of the encapsulated NPs it was not possible to examine their release kinetics in physiological condition as we have done previously^21^ The optical analyzer was used to detect any change in light scattering due to the variation of the size of the nanocapsules or any leaking free NCs or their volume fraction by monitoring the kinetics of destabilization of the obtained systems with encapsulated NaYF_4_:Tm^3+^,Yb^3+^ NPs (see Fig. 2) over a period of 14 days. The example BS profiles of the selected PE nanosuspensions (system 1a) on day 0 (freshly prepared), and after 14 days of storage are shown in Fig. 4a, while in Fig. 4b we present the BS changes in the center of the tube in time for all the studied samples from Table 1.

The *∆*BS parameter was defined in the way similar to that reported in^27^ as the difference in mean BS value between the first scan and n^th^ scan. From Fig. 2 it is found that the surface modification of NaYF_4_:Tm^3+^,Yb^3+^ NPs caused by the LbL adsorption of PEs on the charged oil-cores creates a repulsive force between the particles, which stabilizes the encapsulated NPs. There is practically no evolution in back-scattering of most analyzed nanocapsules (systems 1a, 1b, 1d and 2a, 2b, 2d), which means that no particle destabilization occurred over the analyzed period. Nevertheless, the sedimentation of the large particles in systems 1c and 2c was observed, since in those samples the total weight of nanocapsules and NPs overcomes the repulsive forces. The sedimentation phenomenon is, however, rather minor, which could indicate that the encapsulated NaYF_4_:Tm^3+^,Yb^3+^ are stable also at higher NPs concentration. The influence of the repulsive force in BS measurements on inorganic material stabilization was previously described for gold nanoparticles modification by ionic surfactants and correlated with the different NPs weight, surface charge and size^28^. Thus, we have also correlated the provided BS measurements with the negligible variations of the surface charge, hydrodynamic diameter and polydispersity for all samples after their storage for 30 days (Table 1S) indicating stability of the obtained nanosystems.

### Up-converting luminescence properties of as-synthesized and encapsulated NaYF_4_:Tm^3+^,Yb^3+^ NPs

After assuring the excellent colloidal character of the obtained nanocapsules, and before biological evaluation, we have investigated their optical properties. Upon NIR light (980 nm) excitation the as-synthesized NaYF_4_:Tm^3+^,Yb^3+^ NPs in chloroform and NPs–loaded nanocapsules showed intense luminescence in the visible and NIR spectral regions (Fig. 5). The emission peaks centered at approximately 480 nm, 650 nm and 800 nm were assigned to the ^1^D_2_→^3^F_4_ + ^1^G_4_→^3^H_6_, ^1^G_4_→^3^F_4_, ^3^H_4_→^3^H_6_ electronic transitions in Tm^3+^ ions, respectively. Favorably for biological studies, an intense NIR emission at 800 nm was observed, and thus efficient NIR to NIR bioimaging could be performed with the obtained nanostructures.

Based on the up-converted emission intensities measurements for all of the investigated samples three general trends were observed: (i) the overall emission intensity increased with higher NaYF_4_:Tm^3+^,Yb^3+^ NPs concentration inside the nanocapsules, however, (ii) the relative intensity of the emission bands remained almost unchanged (Fig. 5b,c). Additionally, (iii) the type of the used PE coating did not strongly influence the emission intensity nor its spectral shape. The used encapsulation process and polyelectrolyte LbL assembly preserved the initial ligand coating and hydrophobic environment of NaYF_4_:Tm^3+^,Yb^3+^ NPs inside the nanocapsules, and thus the destructive influence of water vibrational modes on Tm^3+^ ions emission appeared to be minimalized. Nevertheless, the up-converted Tm^3+^ ions emission was shown to be less prone to water molecules quenching than the corresponding visible emission from Er^3+^ ions. Arppe *et al*.^29^ reported up to 60% decrease of Tm^3+^ ions emission intensity, and up to 20% decrease of the corresponding excited states lifetimes related to the NPs + water interactions. The protective role of nanocapsules was in our case also seen in time domain measurements. We have measured the decay curves for emission centered at 480 nm and 800 nm, and the calculated lifetime values are collected in Table 2, while the representative curves are presented in Fig. 6.

**Figure 3:**
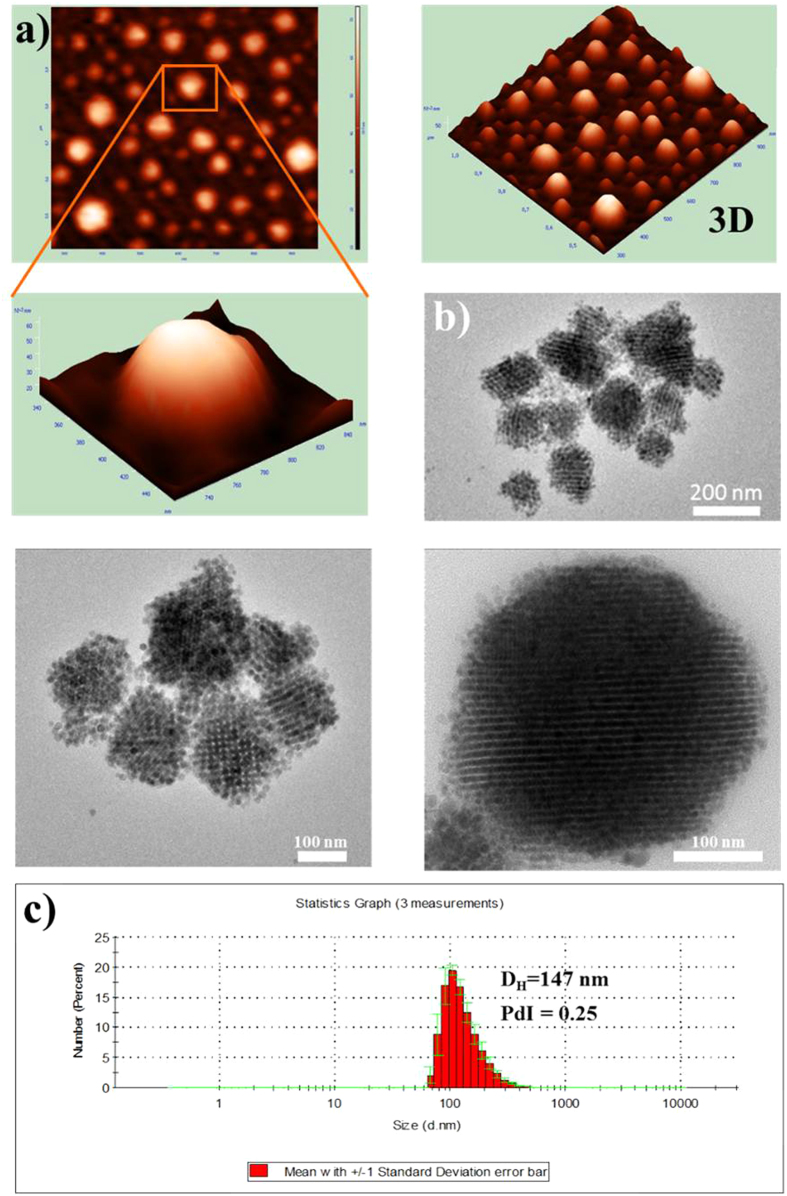
Morphology characterization of encapsulated NaYF_4_:Tm^3+^,Yb^3+^ NPs. Representative AFM (**a**) and TEM (**b**) images of NaYF_4_:Tm^3+^,Yb^3+^ NPs loaded in C_12_(DAPACl)_2_/SO/water (DEX/CHIT) nanocapsules compared to the DLS (**c**) size distribution graph (system 2c according to Table 1).

**Figure 4:**
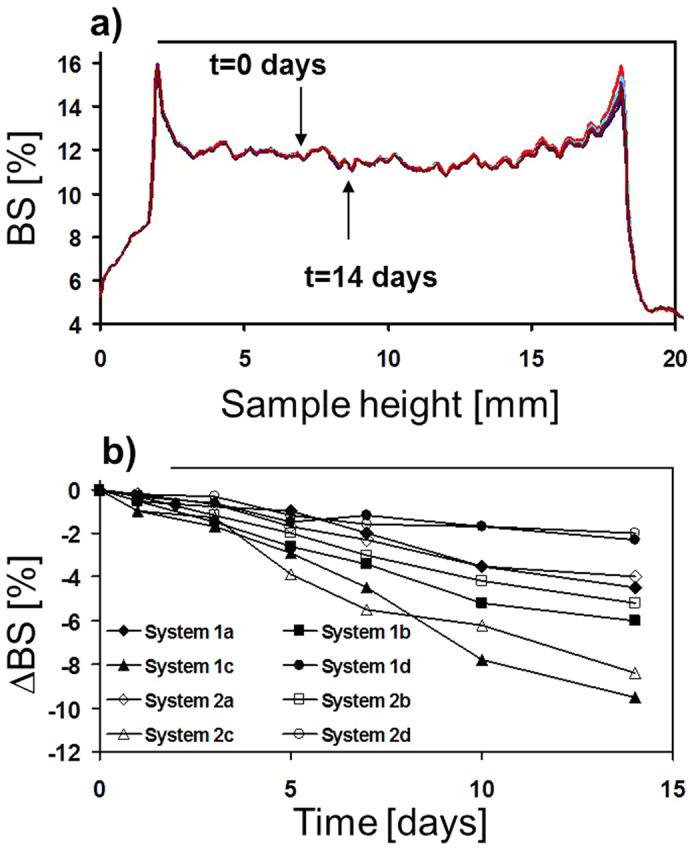
Backscattering (BS) profiles along the loaded nanocapsules as a function of sample height (mm) and time for system 1a (**a**) as well as the same experiment, expressed as ∆BS (referred to the first measurement) for all the studied systems from Table 1 (b).

**Figure 5:**
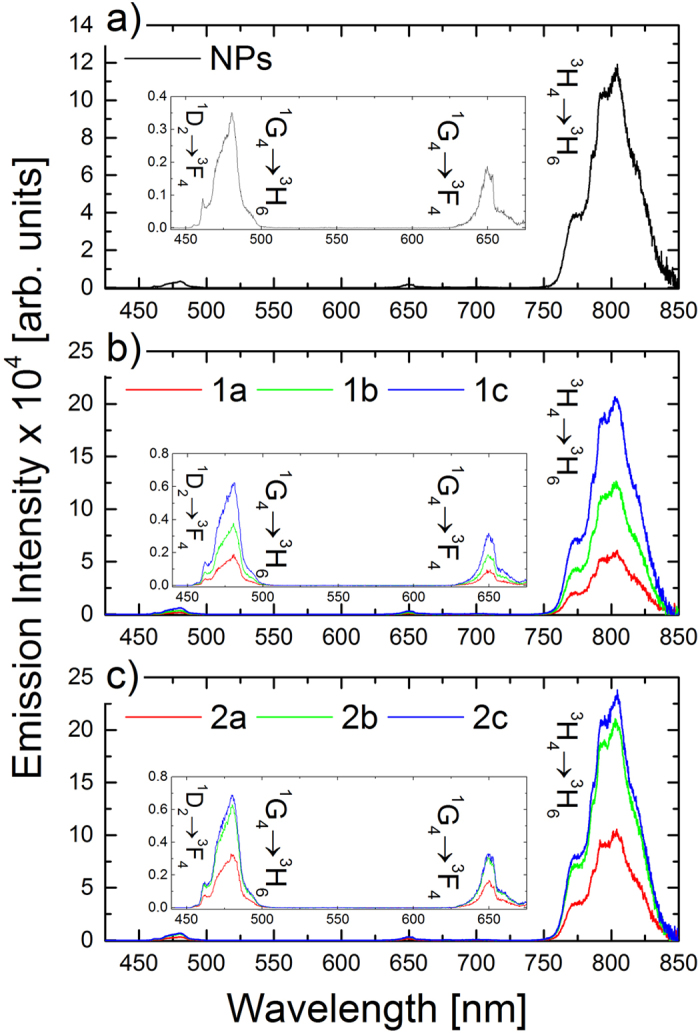
Up-converted emission spectra of as-synthesized NaYF_4_:Tm^3+^,Yb^3+^ NPs in CHCl_3_ (**a**) and after loading in PSS/PDADMAC (**b**) and DEX/CHIT (**c**) nanocapsules with different concentrations under 980 nm laser diode excitation.

**Figure 6:**
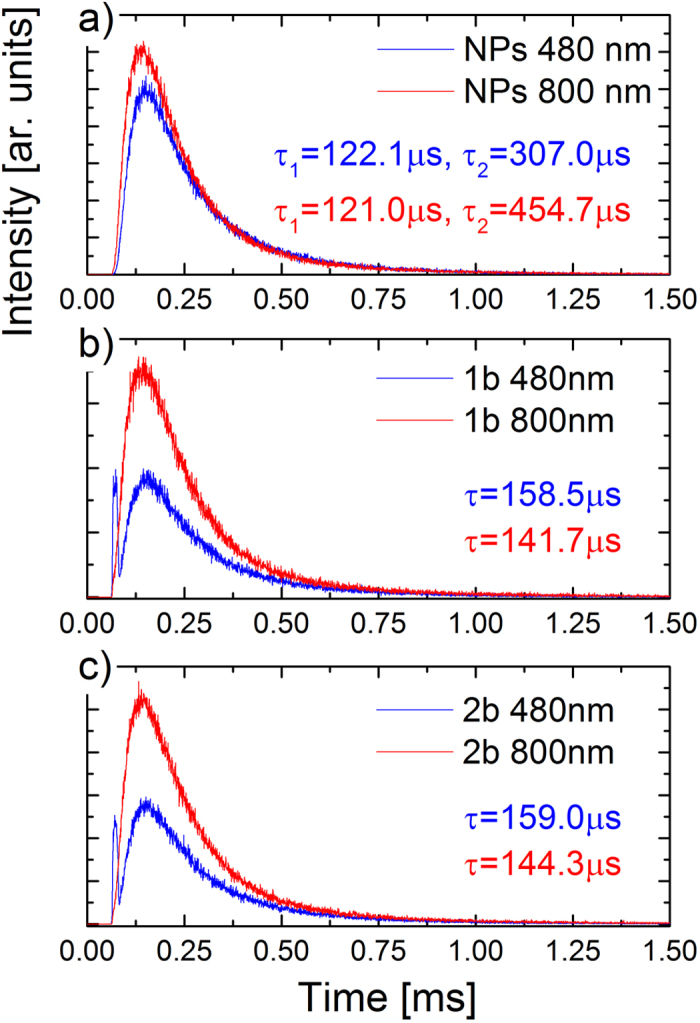
Representative luminescence decay curves, after 980 nm pulsed excitation, of as-synthesized NaYF_4_:Tm^3+^,Yb^3+^ NPs in CHCl_3_ (**a**) and after loading in PSS/PDADMAC (**b**) and DEX/CHIT (**c**) nanocapsules.

All of the studied samples showed long luminescence lifetime values, characteristic for 2% of Tm^3+^ and 20% of Yb^3+^ ions co-doped NaYF_4_ NPs^30^,^31^. For the NaYF_4_:Tm^3+^,Yb^3+^ NPs loaded in nanocapsules, we have observed the change from double to single exponential character of luminescence kinetics and also a slight increase of both blue and NIR emission luminescence lifetime values (Table 2), which can be most probably related to the change of surrounding solvent from chloroform to oil, as well as self-assembly and structuring of NPs inside the nanocapsules as seen in TEM images (Fig. 3). The great importance of the proposed approach for UCNPs adaptation for bio-related applications stems mostly from the unchanged surface of the individual particles. The evident lack of surface functionalization and solvent related quenching, seen in luminescent lifetime measurements, assure the preserved high up-converted emission generation efficiency of the obtained nanocarriers.

### Cytotoxicity of nanocapsules loaded by NaYF_4_:Tm^3+^,Yb^3+^ NPs

**Figure 7:**
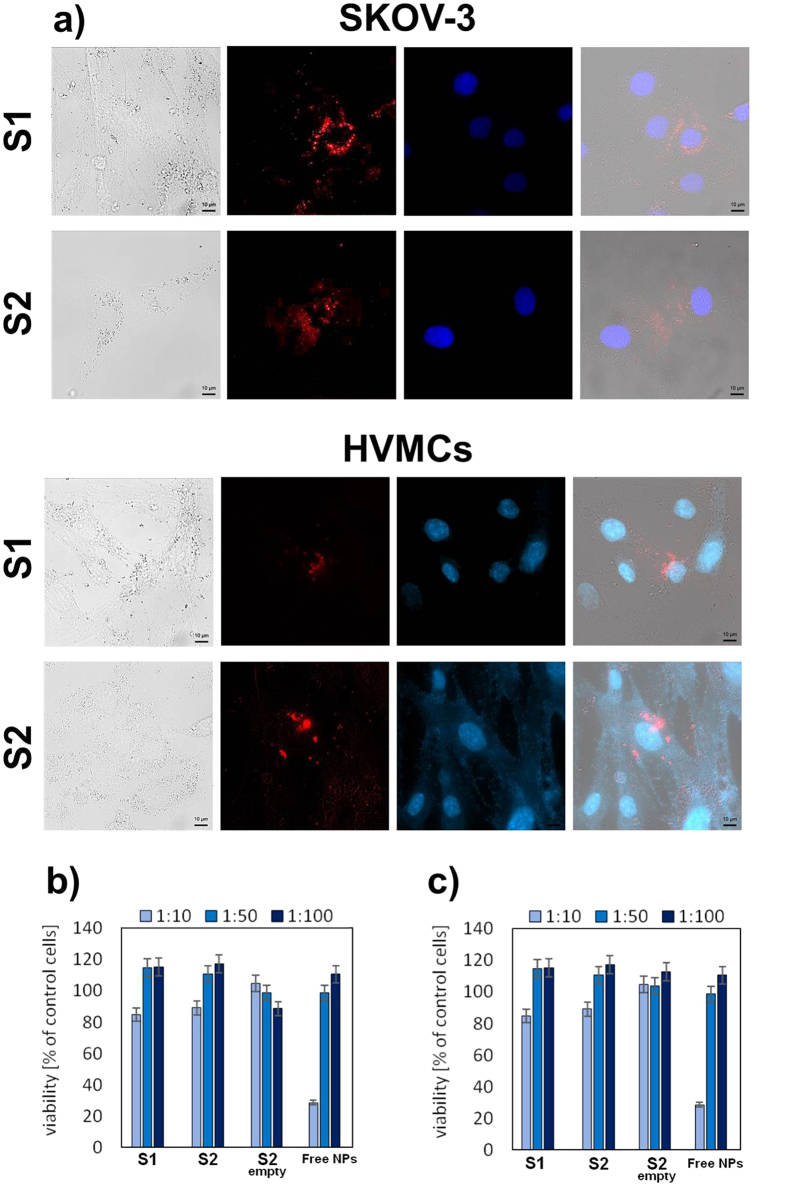
Fluorescence microscopy imaging of human ovarian (SKOV-3) cell line and human vaginal mucosal cells (HVMCs) after incubation with nanocapsulated up-converting NaYF_4_:Tm^3+^,Yb^3+^ NPs for 4 h (**a**) gray: cell in bright field, blue: DAPI (nuclear staining), red: the up-converted emission from NPs and overlay of the both images. Cell viability data of SKOV-3 cells (**b**) and HVMCs (**c**) evaluated by the standard MTT assay for the both PSS/PDADMAC and DEX/CHIT nanocapsules (system 1b and 2b from Table 1, marked as S1 and S2 respectively) after incubation for 24 h.

While the multilayer nanocapsules loaded with NaYF_4_:Tm^3+^,Yb^3+^ NPs showed good water solubility and high colloidal stability, together with high up-converted luminescence intensity and long luminescence lifetimes, it is necessary to investigate the cytotoxicity of the nanocarriers, because the toxicity can be attributed to several factors such as concentration, hydrodynamic size, surface charge and the route of administration^32^. The encapsulated NaYF_4_:Tm^3+^,Yb^3+^ NPs-induced cytotoxic effects on well characterized human cancer cell line - ovarian carcinoma (SKOV3), as well as on normal human vaginal mucosal cells (HVMCs) were evaluated by MTT assays as shown in Fig. 7. The cellular viabilities were estimated after 24 h incubation for the both PSS/PDADMAC and DEX/CHIT nanocapsules, (system 1b and 2b from Table 1, marked in Fig. 7 as S1 and S2 respectively), after incubation for 24 h. Our *in vitro* cytotoxicity study confirmed that the NaYF_4_:Tm^3+^,Yb^3+^ NPs-loaded multilayer nanocapsules, in contrast to the free NPs, exhibit low cytotoxicity upon the both studied human cell lines even at higher nanocapsules concentration (survival rate of 80% relative to control). It should be also mentioned that empty nanocarriers did not cause any decrease in cell survival and there were no major differences in the protective effect of the nanocarriers with different, i.e. standard (PSS/PDADMAC) and sugar-based (DEX/CHIT), PE coatings showing the nanosystems potential for cell imaging and *in vivo* intravenous or direct vaginal administration.

### Bioimaging in normal and cancer cells

The novel nanocontainer design presented here makes the NaYF_4_:Tm^3+^,Yb^3+^ NPs-loaded multilayer nanocapsules attractive biocompatible alternatives for up-conversion cell imaging as luminescent biological labels. We conducted *in vitro* biological experiments using HVMCs and SKOV3 cells to demonstrate the potential of the obtained nanocontainers as probes for bioimaging, and to determine the nanocapsules location inside the cells. Fig. 7a displays the fluorescent images with 409 nm (DAPI) and 975 nm (NaYF_4_:Tm^3+^,Yb^3+^ NPs) excitation, respectively, together with the overlay of both images. The blue regions visible in the pictures are derived from cell nuclei stained with a dye molecule (DAPI), and the red regions (false color) are ascribed to Tm^3+^ ions emission under 976 nm laser diode excitation. In order to prove the lack of interaction between the hydrophobic core of nanocapsules and the cells, we have compared the up-converted emission spectra of dried nanocapsules solution with the corresponding ones taken from the cells (Fig. S4), and no evident changes in the intensity ratio between emission bands were observed. Comparing the nuclei staining and the bright field observation, our results indicate that the cell morphology was unaffected by nanocontainers. It was found from fluorescent microscopy analysis that nanocapsules entered into both cell lines, and the red fluorescent signal was distributed mainly in cytoplasm region (observed as red spots) rather than the nucleus region. Our results are consistent with the other investigations describing the cytoplasmic localization of up-converting NaYF_4_ NPs applied in anticancer therapy^33^. Kamińska *et al*. obtained similar microscopic images of non-functionalized nanoparticles incubated with HeLa cells. They used autofluorescence of cancer cells to detect the potential localization, suggesting that nanoparticles were endocytosed^34^. There was also observed stronger intracellular distribution in ovarian cancer cells. In our study it is also clear that both types of the multilayer nanocarriers are internalized more effectively by tumor ovarian cells in comparison to the normal vaginal mucosal cells, which confirms the protective effect of the applied nanocontainers. The more efficient uptake of the nanocontainers by cancer cells suggests their potential to be used as nanoprobes in such cells, performing a multitude of tasks as imaging for diagnostic information and delivery of therapeutic agents. Furthermore, based on slightly more intense signal for the nanocapsules with polysaccharide shell (DEX/CHIT) we can suppose that those nanosystems were more effectively taken by the studied cells than the nanocarriers with the standard PE layer. Our findings are based also on the earlier reports, which have shown that the sugar-based units are sufficiently stable in the blood circulation and extracellular fluid. But, after cell internalization, they will be cleaved rapidly under intracellular reductive conditions^35^. Thus, the proposed nanocontainer structure based on bioreducible polymers could be utilized for *in vivo* cell labelling in organism tissues to study the precise location and influence of foreign materials and also facilitate other kinds of combination with luminescent materials (i.e. cytostatic drugs or photosensitizers) for different anticancer applications.

## Conclusions

We have designed, engineered and characterized a new type of stable, monodisperse and biocompatible multifunctional core-shell structured nanocontainer for encapsulation of different types of hydrophobic, inorganic NPs, with potential applications in fluorescence imaging and drug delivery. Our results prove that the emulsification-solvent diffusion approach followed by LbL saturation technique using environmental friendly surfactant leads to efficient stabilization of silicone core polymeric nanocarriers layered by synthetic or natural polyelectrolyte shells. The performed DLS and light BS measurements showed prolonged colloidal stability and lack of evident destabilization phenomena associated with unwanted cargo release or sedimentation of the obtained nanocapsules. Furthermore, these nanostructures exhibited a low toxicity against both human ovarian carcinoma (SKOV3) and normal human vaginal mucosal cells (HVMCs), even at high nanocapsules concentrations. While the structuring of the polymeric nanocapsules ensured the colloidal stability, absence of inherent toxicity and targeting properties, the cargo of lanthanide-doped up-converting NPs provided favorable luminescence properties. Using Tm^3+^ and Yb^3+^ co-doped NaYF_4_ NPs as fluorescence markers gave the possibility to perform stable in time, NIR to NIR bioimaging, with significantly reduced autofluorescence background signal. As in the proposed approach the cumbersome functionalization of single NPs surface was omitted, and NPs preserved their initial ligand coating and hydrophobic environment inside the nanocapsules, the high up-conversion emission performance was observed, with no evident destructive quenching. Finally, the obtained nanocarriers with sugar based shielding showed favorable internalization and bioimaging ability in the tumor cells, which proves their high specificity and selectivity. Our results provide a new and exciting insight into developing methods of construction of biocompatible and multifunctional nanoscale structure taking advantage of intact luminescent properties of up-converting NPs with simultaneous low cytotoxicity and biologically important selectivity.

## Materials and Methods

### Reagents

Most of the starting materials were purchased from Sigma Aldrich or POCH and used as received. 3,3′-iminobis(*N,N*-dimethylpropylamine) and lauroyl chloride were purchased from Aldrich Chemical Co. (Milwaukee, WI), hydrochloric acid was purchased from POCH (Gliwice, Poland). Other reagents and solvents were of commercial grade and were not additionally purified before use. Nuclear magnetic resonance (^1^H NMR) spectra were recorded for solutions of samples in CDCl_3_ using a Bruker Avance DRX300 spectrometer (Bruker, Karlsruhe, Germany). Silicone oil (SO, from Stearinerie Dubois Fils, France) was used as the oil phase. Poloxamer 403 (polymer) was obtained from BASF (Ludwigshafen, Germany). Polyelectrolytes: Chitosan (medium molecular weight), dextran sulfate sodium salt (*M*w ~40 000), poly(diallyldimethylammonium) chloride (PDADMAC, *M*w ~100 000–200 000, 20% in water), and polysodium 4-styrenesulphonate (PSS, *M*w = 70 000) were obtained from Sigma-Aldrich. Water used for all experiments was doubly distilled and purified by means of a Millipore (Bedford, MA) Milli-Q purification system.

### Synthesis of up-converting NaYF_4_:Tm^3+^,Yb^3+^ NPs

The ~8 nm in size NaYF_4_ NPs doped with 2% of Tm^3+^ and 20% of Yb^3+^ were synthesized based on protocol reported by Chen *et al*^23^. Briefly, 1 mmol of lanthanide oxides (Tm_2_O_3_, Yb_2_O_3_, and Y_2_O_3_) and 2 mmol of NaOH were mixed and dissolved in 50% concentrated trifluoroacetic acid (10 mL) at 95 °C in a 250 mL three-neck flask, and the transparent solution was evaporated using rotary evaporator to dryness. Next, 16 mL of oleic acid and 8 mL of oleylamine were added into the flask. The solution was then heated to 120 °C under vacuum with magnetic stirring for total oxygen and water removal, during which the flask was purged with dry argon every 10 min. The resulting yellow solution was then heated to 275 °C under inert atmosphere and kept at these conditions for 30 min. The final mixture was cooled to room temperature, precipitated by acetone, washed several times with ethanol and collected by centrifugation at 10,000 rpm for 15 min. The final stock solution of as synthesized NaYF_4_:Tm^3+^,Yb^3+^ NPs was dispersed in 5 mL of chloroform at the concentration of ~98 mg/mL.

### Synthesis of *N,N*-bis[3,3-(dimethylamine)propyl] dodecanamide dichloride: general procedure according to our previous method

*N,N*-bis[3,3-(dimethylamine)propyl] dodecanamide dichloride, C_12_(DAPACl)_2_ was synthesized by reaction of stoichiometric amounts of dodecanoyl chloride (14.0 g, 0.0641 mol) and 3,3′-iminobis(*N,N*-dimethylpropylamine) (12.0 g, 0.0641 mol) in chloroform solution (150 ml) at room temperature in the presence of saturated NaHCO_3_ aqueous solution (60 ml). Crude liquid product was isolated by means of extraction and carefully purified by conversion to crystalline hydrochloride and repeated crystallization from acetone/chloroform mixture^36^. The yield of the final product was above 75%. Purity and chemical structures were proved by analysis of ^1^H NMR spectra. ^1^H NMR *δ*_H_ (300 MHz, CDCl_3_, Me_4_Si): 0.86–0.90 (3H, t, *CH*_3_(CH_2_)_8_CH_2_CH_2_CON-); 1.26–1.29 (16H, m, CH_3_(*CH*_2_)_8_CH_2_CH_2_CON-); 1.58–1.63 (2H, m, CH_3_(CH_2_)_8_*CH*_2_CH_2_CON-); 2.15–2.29 (4H, m, N[CH_2_*CH*_2_CH_2_N(CH_3_)_2_]_2_); 2.30–2.35 (2H, t, CH_3_(CH_2_)_8_CH_2_*CH*_2_CON-); 2.82–2.84 and 2.91–2.92 (12H, d, N[CH_2_CH_2_CH_2_*N*(*CH*_3_)_2_]_2_); 3.02–3.08 and 3.13–3.20 (4H, t, N[CH_2_CH_2_*CH*_2_N(CH_3_)_2_]_2_ and 3.53–3.61 (4H, t, N[*CH*_2_CH_2_CH_2_N(CH_3_)_2_]_2_); 12.05–12.30 (2H, s, N[CH_2_CH_2_CH_2_N^+^*H*(CH_3_)_2_]_2_).

### Encapsulation of up-converting NaYF_4_:Tm^3+^,Yb^3+^ NPs in multilayer nanocapsules

For the NPs surface hydrophilization we used the emulsification-solvent diffusion method followed by layer-by-layer (LbL) saturation technique proposed by our group^37^,^17^. In the first phase for the polymeric template preparation 4.6 mL of dichloromethane solution consisting of the fixed quantities of the silicone oil 1.5% (w/w) and Poloxamer 403 3% (w/w), was mixed with 200–800 μL of NaYF_4_:Tm^3+^,Yb^3+^ NPs (0.4–1.5 mg/mL dissolved in chloroform) under magnetic stirring (30 min at 25 °C). Afterward, the solutions were sonicated for 90 seconds and transferred to a rotary evaporator (Buchi Rotavapor R-200) with a water bath set at 40 °C, under vacuum to remove excess solvents. Next, 5 mL of aqueous solutions containing an environmental friendly cationic dicephalic-type surfactant, i.e., N,N-bis[3,3-(dimethylamine)propyl] dodecanamide dichloride, C_12_(DAPACl)_2_ was added dropwise to the evaporated mixture with water bath set at 40 °C at vigorous magnetic stirring for 1 h. The obtained suspension was then sonicated for 5 min, and the final NPs-loaded polymeric nanocarriers were cooled at ambient temperature overnight. In the last step, the relevant suspension was separated via nanofiltration process using poly(ether sulfone) (PES) membranes (pore size 200 nm). In the second phase two types of polyelectrolyte (PE) coatings: sugar-based (cationic chitosan, CHIT and anionic dextran, DEX) and standard (anionic polysodium 4-styrenesulphonate, PSS and cationic poly(diallyldimethyl-ammonium) chloride, PDADMAC) were used for long sustained oil-core multilayer nanocapsules formation on the polymeric template obtained previously by an emulsification-solvent diffusion method. Solutions of the selected PE’s (of concentration equal to 1 g/L) were pre-pared by dissolving each polyion in NaCl solution of ionic strength 0.015 M. To improve solubility of CHIT, pH of the PE solution was adjusted by addition of HCl to pH 2. Solutions of the PDADMAC, PSS and DEX were prepared without any pH adjustment. The LbL deposition was performed using the saturation method without the intermediate rinsing step during continuous mixing with a magnetic stirrer at 300 rpm. Volumes of polyelectrolyte solution used to form each layer were chosen experimentally by assessing the results of simultaneous zeta potential measurements.

### General characterization methods of NaYF_4_:Tm^3+^,Yb^3+^ NPs

The morphology of as synthesized and encapsulated NaYF_4_:Tm^3+^,Yb^3+^ NPs was studied with a FEI Tecnai G^2^ 20 X-TWIN transmission electron microscope (TEM), and the crystal structure of as synthesized NPs with a STOE diffractometer with Ge-filtered CuK_α1_ radiation. The spectroscopic properties of colloidal solutions of both as-synthesized and encapsulated NaYF_4_:Tm^3+^,Yb^3+^ NPs, including up-converted emission spectra, ^1^G_4_ and ^3^H_4_ energy level lifetimes, were measured with an Edinburgh Instruments FLS 980 spectrofluorometer after excitation with fractionated 980 nm laser diode (8W, Spectra-Laser, Poland).

### Characterization methods of multilayer polymeric nanocarriers

#### Zeta potential (ζ-potential)

The particle charge (*ζ*-potential) of the nanocapsules was measured by the microelectrophoretic method using a Malvern Zetasizer Nano ZS apparatus. All the measurements were performed at 25 °C. Each value was obtained as an average of three subsequent runs of the instrument with at least 20 measurements.

#### Dynamic light scattering (DLS)

The size distribution, (i.e. the hydrodynamic diameter, D_H_) of the nanocapsules was determined by DLS using the Zetasizer Nano Series from Malvern Instruments with the detection angle of 173° in optically homogeneous square polystyrene cells. All measurements were performed at 25 °C. Each value was obtained as an average of three runs with at least 10 measurements. The DTS (Nano) program was applied for data evaluation.

#### Multiple light scattering measurements

Long-term physical stability of the nanocapsules was assessed using a TurbiScanLab (Formulaction SA, France) by measuring the backscattering (BS) of pulsed near infrared light (λ = 880 nm). Two synchronous optical sensors receive, respectively, light transmitted through the sample (0° from the incident radiation, transmission sensor), and light back-scattered by the sample (135° from the incident radiation, backscattering detector). The sample in a cylindrical glass cell was scanned at 25 °C while moving along the entire height of the cell and BS profiles as a function of sample height were collected and analyzed using the instrument’s software (Turbisoft version 2.0.0.33). The same measurements were performed for freshly prepared nanocapsules and repeated on the same samples after 14 days of their storage at 25 °C.

#### Atomic force microscopy (AFM)

The morphology of the nanocapsules was investigated using a Veeco NanoScope Dimension V AFM with an RT ESP Veeco tube scanner. The scanning speed was 0.5 Hz and a low-resonance-frequency pyramidal silicon cantilever resonating at 250–331 kHz was employed (at a constant force of 20–80 N/m). The amplitude of the resonance was set manually to the lowest possible value for stable imaging within the contamination layer present on the surface. Before observations, the nanocapsules were allowed to adsorb on freshly cleaved mica surface for 12 hours by dipping it in the suspension. Then, the excess of substrate was removed by rinsing the mica plates in double distilled water for 15 minutes and drying at room temperature.

### Ethics Statement

Cell studies, protocols and isolation procedures were carried out in accordance with the ethics guidelines under Polish national rules and according to the principles of the Declaration of Helsinki, and were approved by the Bioethics’ Committee of Wroclaw Medical University (Poland) (dispatch 26, Nov 2014). Informed consent was given by the patient involved in the study and collected according to guidelines from the fields of medical ethics and research ethics. All documentation is stored in the hospital archives. The study did not imply collection of extra material from the healthy female donor (only vaginal mucosal tissues were used). The study did not bring any direct benefits to the volunteer; there were no risks or costs for the volunteer; there was no access to patient clinical data (samples were obtained in anonymous form from the Specialized Hospital of Antoni Falkiewicz in Wroclaw (Poland)); participation was volunteer and could be interrupted at any time; there are no ethical impacts predicted; there will be no commercial interests.

### Cell culture and MTT assay for cytotoxicity

The studies were performed on well characterized human cancer cell line – ovarian carcinoma resistant to diphtheria toxin, cisplatin and adriamycin (SKOV-3) - as well as on normal human vaginal mucosal cells (HVMCs). The HVMCs were obtained from healthy women approved by the Bioethics Committee of Specialized Hospital of Antoni Falkiewicz in Wroclaw (Poland). The HVMCs isolation procedure was performed according to the protocol described in the patent^38^. The SKOV-3 cells were derived from ATCC (HTB-77). The cells were cultured and prepared to the experiments according to the conditions described previously^39^,^40^. The *in vitro* cytotoxicity evaluation of the studied nanocarriers loaded with NaYF_4_:Tm^3+^,Yb^3+^ NPs (C_NPs_ = 0.9 mg/mL) in three different dilutions (1:10, 1:50, 1:100) was performed using a MTT reduction assay, after 24 h of the cell treatment according to the manufacturer’s protocol^22^. The cellular viabilities were estimated for both PSS/PDADMAC and DEX/CHIT nanocapsules, (system 1b and 2b from Table 1, marked in Fig. 7 as S1 and S2 respectively). The cell viability in each group was expressed as a percentage of control (untreated with studied nanoparticles) cells. The data were obtained by calculating the average of three experiments.

### *In vitro* cells imaging

The imaging experiments were performed on a fully automated (Carl Zeiss AxioObserver Z1) inverted fluorescent microscope equipped with definite focus for the Z drift elimination and operated by the ZEN software. Image detection was performed by a high sensitivity and high speed EMCCD (Rolera EM-C^2^) camera, while the spectra measurements was done with a Shamrock SR303i spectrograph (Andor) and a cooled, high-sensitivity scientific CMOS (sCMOS) sensor (Newton 1600×400 back-thinned). The bright field images were taken with use of a halogen lamp source and the Zeiss LCI Plan – NEOFLUAR x60 oil immersion objective. The organic dye (DAPI) blue emission excited by the metal-halide lamp was detected with a DAPI long-pass filter cube composed of Semrock 377/50 nm BrightLine single-band bandpass filter for the excitation, 409 nm edge BrightLine single-edge dichroic beam splitter and the 409 nm blocking edge BrightLine long-pass filter. Encapsulated up-converting NaYF_4_:Tm^3+^,Yb^3+^ NPs were excited by a 975 nm laser diode at ca. 1.6 W of optical power measured at the laser diode output. The laser diode was coupled to the rear port of the microscope by a custom built optical setup allowing for an automatic switching between different excitations. The up-converted emission was detected with use of a custom built filter cube composed of the Semrock 757 nm edge BrightLine single-edge dichroic beam splitter and the 945 nm blocking edge BrightLine short-pass filter.

## Additional Information

**How to cite this article**: Bazylińska, U. *et al*. Polymeric nanocapsules with up-converting nanocrystals cargo make ideal fluorescent bioprobes. *Sci. Rep.* **6**, 29746; doi: 10.1038/srep29746 (2016).

## Supplementary Material

Supplementary Information

## Figures and Tables

**Table 1:** Characteristics of NaYF_4_:Tm^3+^,Yb^3+^ NPs loaded nanocapsules with synthetic or natural PE shell prepared by an emulsification-solvent evaporation method followed LbL approach.

System	Composition/PE bilayer type	C_NPs_(mg/ml)	D_H,_ (nm)^a^	PdI^b^	ζ (mV)^c^
1a		0.4	126 ± 4	0.19 ± 0.02	+55 ± 7
1b	C_12_(DAPACl)_2_/Pol 403/SO/water (PSS/PDADMAC)	0.9	126 ± 4	0.24 ± 0.03	+56 ± 8
1c		1.5	143 ± 7	0.23 ± 0.03	+56 ± 8
1d		empty	110 ± 3	0.23 ± 0.03	+57 ± 8
2a		0.4	130 ± 5	0.22 ± 0.02	+41 ± 6
2b	C_12_(DAPACl)_2_/Pol 403/SO/water (DEX/CHIT)	0.9	136 ± 6	0.24 ± 0.03	+40 ± 5
2c		1.5	147 ± 8	0.25 ± 0.04	+43 ± 6
2d		empty	114 ± 3	0.23 ± 0.03	+40 ± 5

^a^Hydrodynamic diameter.

^b^Polydispersity index.

^c^Zeta potential, mean ± SD, n = 3.

**Table 2:** Luminescence lifetime values of as-synthesized NaYF_4_:Tm^3+^,Yb^3+^ NPs and loaded in nanocapsules with synthetic or natural polyelectrolyte shell prepared by LbL approach.

System	Composition/PE bilayer type		C_NPs_(mg/ml)	LT 480 nm [μs]	LT 800 nm [μs]
NPs			—	τ_1_ = 122.1	τ_1_ = 121.0
	NaYF_4_:Tm^3+^,Yb^3+^		τ_2_ = 307.0	τ_2_ = 454.7	
1a	C_12_(DAPACl)_2_/Pol 403/SO/water (PSS/PDADMAC)	403/SO/water	0.4	τ_1_ = 157.4	τ_1_ = 140.7
1b			0.9	τ_1_ = 158.5	τ_1_ = 141.7
1c			1.5	τ_1_ = 157.5	τ_1_ = 145.3
2a	C_12_(DAPACl)_2_/Pol 403/SO/water (DEX/CHIT)	403/SO/water	0.4	τ_1_ = 157.5	τ_1_ = 141.8
2b			0.9	τ_1_ = 159.0	τ_1_ = 144.3
2c			1.5	τ_1_ = 160.0	τ_1_ = 143.8
