# Supplementary material for: Polymeric nanocapsules with up-converting nanocrystals cargo make ideal fluorescent bioprobes
Source: Sci Rep. 2016 Jul 13;6:29746. doi: 10.1038/srep29746 (PMC4942829; doi:10.1038/srep29746)
Supplement: Supplementary Information [file srep29746-s1.pdf]

Supplementary information (ESI) for

Polymeric nanocapsules with up-converting nanocrystals cargo make ideal fluorescent bioprobes

U. Bazylińska[a], D. Wawrzyńczyk[b], J. Kulbacka[c], R. Frąckowiak[a], B. Cichy[d], A. Bednarkiewicz[d,e], M. Samoć[b*], K. A. Wilk[a*]

[a)] Department of Organic and Pharmaceutical Technology, Faculty of Chemistry, Wrocław University of Science and Technology, Wybrzeże Wyspiańskiego 27, 50-370 Wroclaw, Poland.
E: mail: kazimiera.wilk@pwr.edu.pl

[b)] Advanced Materials Engineering and Modelling Group, Faculty of Chemistry, Wrocław University of Science and Technology, Wybrzeże Wyspiańskiego 27, 50-370 Wroclaw, Poland,
E: mail: marek.samoc@pwr.edu.pl

[c)] Department of Medical Biochemistry, Medical University of Wroclaw, Chałubińskiego 10, 50-368 Wroclaw, Poland

[d)] Institute of Low Temperature and Structure Research, PAS, Okólna 2, 50-422 Wroclaw, Poland

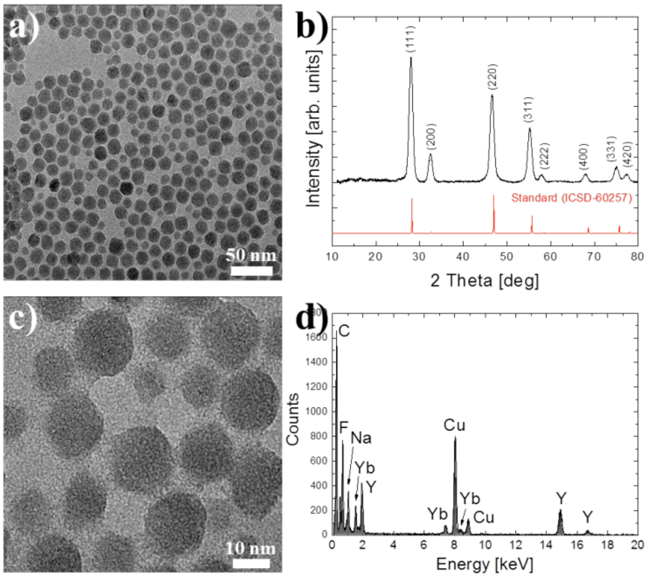

Fig. S1 Characterization of as synthesized $NaYF_4:Tm^{3+},Yb^{3+}$ NPs. TEM (a) and HRTEM (c) images of $NaYF_4:Tm^{3+},Yb^{3+}$ NPs dried from chloroform solutions, together with measured XRD and standard ICSD-60257 patterns (b) and EDS spectrum (d).

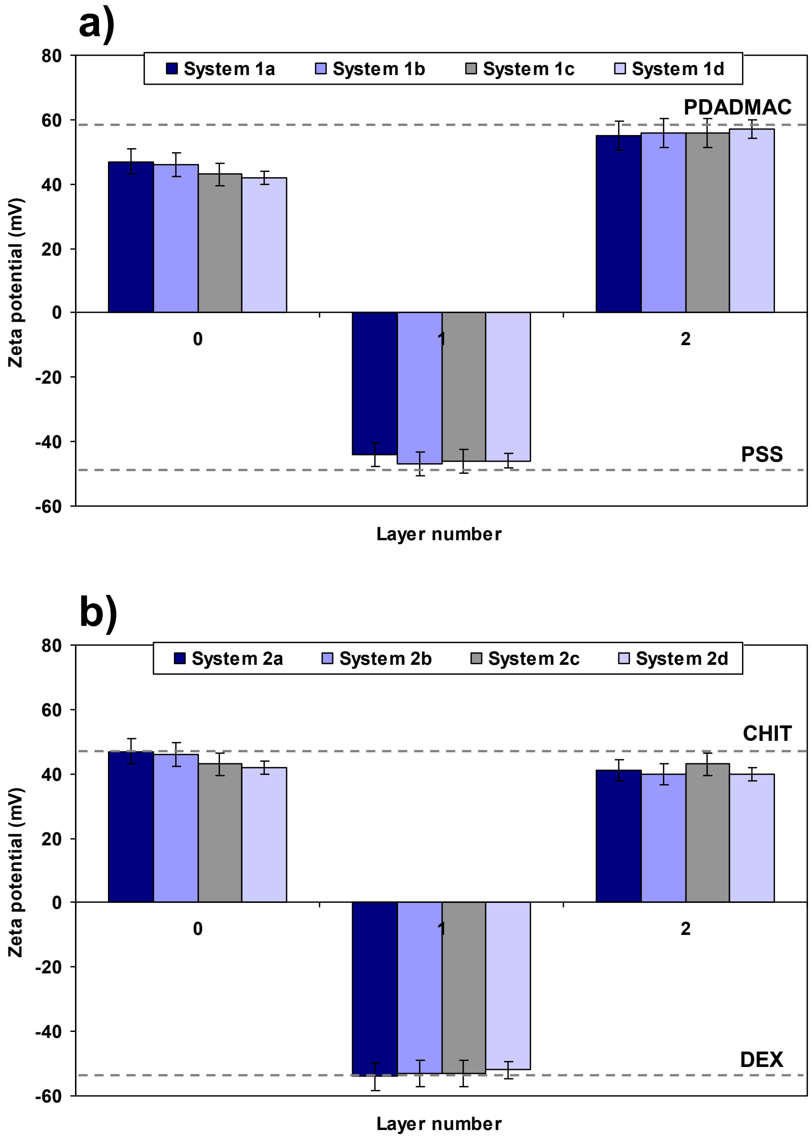

Fig. S2 Layer-to-layer variations of the zeta potential for PSS/PDADMAC (a) and DEX/CHIT coated polymeric template (n=0), empty and loaded by $NaYF_4:Tm^{3+},Yb^{3+}$ NPs (see descriptions for the systems in Table 1).

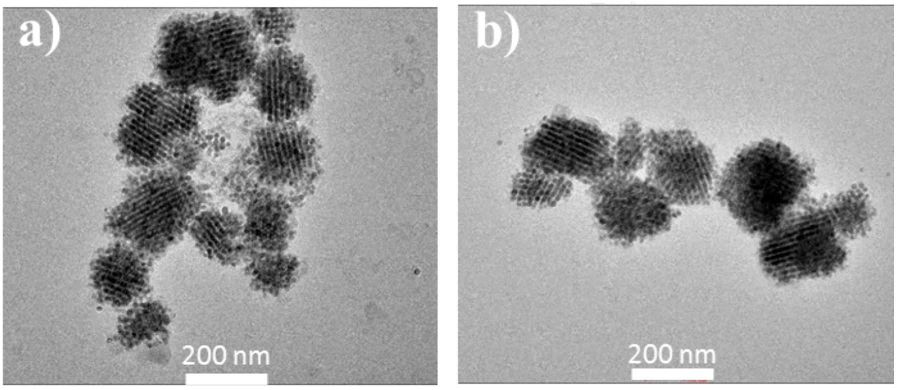

Fig.S3. Representative TEM images of polymeric nanocapsules with $NaYF_4:Tm^{3+},Yb^{3+}$ NPs showing the dispersion of their morphology and sizes.

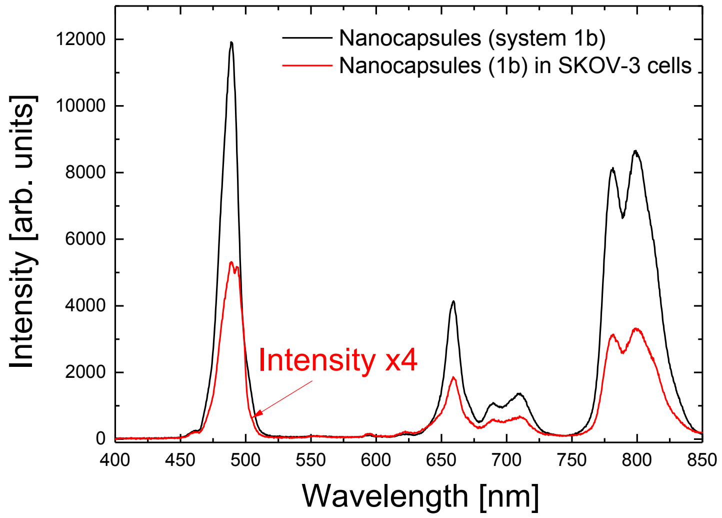

Fig. S4. Representative up-conversion emission spectra of dried solution of nanocapsules (system 1b according to the Table 1) loaded with $NaYF_4:Tm^{3+},Yb^{3+}$ NPs and after incubation with cancer ovarian SKOV-3 cells.
